# Dr. Henry Denton Nicoll

**Published:** 1908-12

**Authors:** 


DR. HENRY DENTON NICOLL, of New York, died at his home at New Windsor, October 27, 1908, aged 64 years. He was one of the surgeons of the Woman's Hospital, consulting surgeon to various hospitals, and president of the law and order union of the State of New York.

